# Profiling DNA-protein interactions in *Meloidogyne incognita* using dCas9-based affinity purification

**DOI:** 10.1186/s13007-025-01475-5

**Published:** 2026-01-04

**Authors:** Caroline Bournaud, Alwéna Tollec, Etienne G. J. Danchin, Yohann Couté, Sebastian Eves-van den Akker

**Affiliations:** 1 Microbiologie, Adaptation et Pathogénie, UMR5240, Univ Lyon, Université Lyon 1, Bayer SAS, 69622 Villeurbanne, France; 2 Univ. Grenoble Alpes, Inserm, CEA, UA13 BGE, CNRS, CEA, UAR2048 ProFI, 38000 Grenoble, France; 3 Institut Sophia Agrobiotech, INRAE, Université Côte d’Azur, CNRS, 400 routes des Chappes, 06903 Sophia-Antipolis, France; 4 The Crop Science Centre, Department of Plant Sciences, University of Cambridge, Cambridge, CB2 3EA UK

**Keywords:** Second stage juvenile (J2), *Meloidogyne incognita*, Sonication, Chromatin preparation, Non-transformable organism, *In vitro* dCas9-based CAPTURE, Promoter pulldown proteomics, Chromatin protein solubilization

## Abstract

**Background:**

The root-knot nematode *Meloidogyne incognita*, is a highly destructive parasite that manipulates host plant processes through effector proteins, affecting agriculture globally. Despite advances in genomic and transcriptomic studies, the regulatory mechanisms controlling effector gene expression, especially at the chromatin level, are still poorly understood. Gene regulation studies in plant-parasitic nematodes (PPN) face several challenges, including the absence of transformation systems and technical barriers in chromatin preparation, particularly for transcription factors (TFs) expressed in secretory gland cells. Conventional methods like Chromatin Immunoprecipitation (ChIP) are limited in PPN due to low chromatin yields, the impermeability of nematode cuticles, and difficulties in producing antibodies for low-abundance TFs. These issues call for alternative approaches, such as dCas9-based CAPTURE (CRISPR Affinity Purification in siTU of Regulatory Elements) that allows studying chromatin interactions by using a catalytically inactive dCas9 protein to target specific genomic loci without relying on antibodies.

**Results:**

This study presents an optimized in vitro dCas9-based CAPTURE for second stage juvenile (J2) *M. incognita* that addresses key challenges in chromatin extraction and stability. The protocol focuses on the promoter region of the *6F06* effector gene, a critical gene for parasitism. Several optimizations were made, including improvements in nematode disruption, chromatin extraction, and protein-DNA complex stability. This method successfully isolated chromatin-protein complexes and identified four putative chromatin-associated proteins, including BANF1, linked to chromatin remodelling complexes like SWI/SN.

**Conclusion:**

The optimized in vitro dCas9-based CAPTURE protocol offers a new tool for investigating chromatin dynamics and regulatory proteins in non-transformable nematodes. This method expands the scope of effector gene regulation research and provides new insights into *M. incognita* parasitism. Future research will aim to validate these regulatory proteins and extend the method to other effector loci, potentially guiding the development of novel nematode control strategies.

**Supplementary Information:**

The online version contains supplementary material available at 10.1186/s13007-025-01475-5.

## Background

*Nematode parasitism and the regulation thereof* Root-knot nematodes (RKN), such as *Meloidogyne incognita*, are among the most destructive plant-parasitic nematodes (PPN), causing significant agricultural losses globally. These nematodes achieve parasitic success by secreting a wide range of effectors - proteins that hijack important host cellular processes (e.g. metabolism, structure, physiology, or immunity), to the benefit of the parasite. These effectors primarily originate from two gland cell types (e.g. two subventral and one dorsal gland), are ultimately injected into host tissues, and are associated with triggering a complex host-pathogen interaction. Over decades, genomic and transcriptomic analyses have substantially advanced our understanding of nematode effector biology [1–6]. Numerous developmental stages transcriptomes across evolutionary distinct plant parasitic nematodes indicate concerted and dynamic expression patterns of effector genes throughout the nematode life cycle, suggesting shared and precise regulatory control [1, 7–9]. Consistent with this global view, a small subset of transcription factors have been found to be strongly co-regulated with effectors over time [10–12]. Notably, a recent study in the cyst nematode *Heterodera schachtii* established a link between a transcription factor, termed subventral gland regulator-1 (SUGR-1), and expression activation of effector genes. This transcription factor, along with other highly connected regulators, is expressed in secretory gland cells and appears to be influenced by both nematode biology and plant-derived signals [10]. Despite recent advances, the regulatory mechanisms controlling effector gene expression in PPN remain poorly understood. Developing DNA-protein interaction approaches will be key to uncovering major regulators of parasitism genes and advancing functional genomics in plant-parasitic nematodes.

*Technical hurdles for studying gene regulation in PPN* Progress in deciphering gene regulation in PPN has been hampered by major technical constraints. Unlike model nematodes such as *Caenorhabditis elegans*, *M. incognita* lacks efficient transformation systems, limiting the use of advanced genetic tools like CRISPR for in vivo functional studies [13]. Chromatin Immunoprecipitation (ChIP) is a well-established method to study global landscape of protein-DNA interactions and histone modifications at specific genomic loci and under a physiological context. Recently, ChIP based on genome-wide histone immunoprecipitation (IP) has revealed the role of histone modifications in the regulation of virulence in *M. incognita*, underscoring the need for precise methods to investigate chromatin landscapes at the single-locus level, particularly for effector genes [14]. While ChIP has been successfully applied to *C. elegans* and some other nematodes [14–21], its use remains limited in PPN. This technique is inherently constrained when it comes to exploring the complete protein landscape of a specific locus, especially when the associates regulatory factors have not yet been characterized. Additional considerations regarding these methodological limitations are discussed below. These challenges highlight the need for alternative strategies capable of dissecting locus-specific DNA-protein interactions in PPN, particularly in non-transformable systems.

**Fig. 1:**
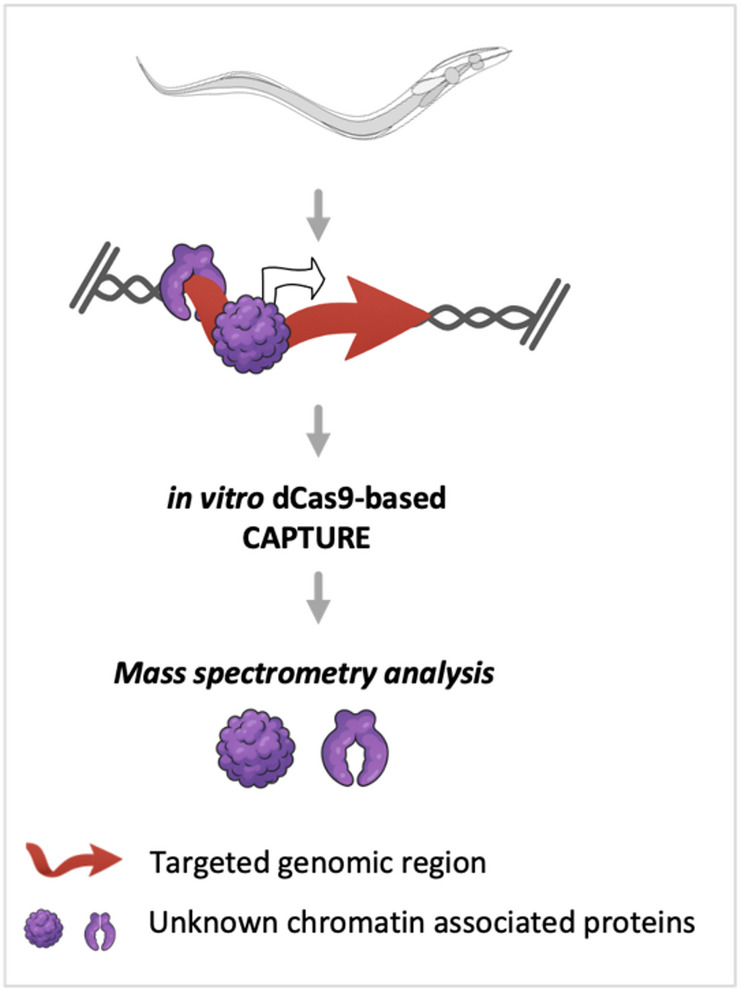
Identification of proteins associated with a specific genomic locus using the in vitro dCas9-based CAPTURE technology. The principle relies on a CRISPR-targeted chromatin-based purification strategy, involving the in vitro use of biotinylated nuclease-deficient Cas9 (dead Cas9) and synthetic guide RNA (gRNA) incubated with fragmented chromatin from the phytonematode *Meloidogyne incognita*. This recombinant ribonucleoprotein complex, coupled with mass spectrometry (MS)-based proteomics, enables the capture and isolation of unknown proteins associated with their genomic region of interest

*dCas9-based CAPTURE: a related ChIP technique for chromatin regulation studies* The dCas9-based CAPTURE protocol offers a promising alternative to conventional ChIP by bypassing the need for specific antibodies (Fig. 1). It uses a catalytically inactive version of Cas9 (dead Cas9 or dCas9 – unable to cleave DNA), guided by single guide RNAs (sgRNAs), to target specific genomic regions without requiring prior knowledge of regulatory proteins [22]. This flexibility allows for the capture of chromatin-protein complexes at specific loci, which can then be identified through mass spectrometry-based proteomics (AP-MS) [22]. The method has been successfully applied in vivo using conventional cell cultures (e.g. cancer cell lines [23]; in *S. cerevisiae* yeast [24]), in in vitro systems (e.g. 293T cells [25]; S2 cells of *D. melanogaster* [26]) and more recently, in multicellular organisms (e.g. plant species *Betula platyphylla* [27]). It has yet to be tested in PPN. Given the non-transformability of *M. incognita* and the challenges associated with traditional ChIP, in vitro dCas9-based CAPTURE provides a viable method for studying chromatin dynamics and regulatory proteins in PPN.

## Objectives and scope of the study

In this study, we optimized an in vitro dCas9-based CAPTURE protocol for second stage juvenile (J2) *M. incognita*, focusing on the promoter region of the *6F06* effector gene. 6F06 is part of a set of early-expressed effector genes that are transcriptionally coordinated, making it a relevant target for investigating chromatin-based regulatory mechanisms in nematode parasitism [1, 14]. Our work addresses key technical challenges related to nematode physical disruption, chromatin extraction, fixation, and fragmentation, ensuring sufficient chromatin yield and stability of protein-DNA complexes. As a proof of concept, we demonstrate the utility of in vitro dCas9-based CAPTURE for investigating chromatin dynamics in non-transformable PPN, and pave the way for future studies on regulatory mechanisms in *M. incognita* and other parasitic nematodes of global concern. This approach aims to expand the current understanding of parasitism and contribute to the development of novel strategies for nematode control.

## Results

Herein we detail the major steps necessary for the implementation of the dCas9-based CAPTURE method to identify DNA-associated proteins at a chosen genomic locus of interest in *M. incognita*. To reach this goal, we focused on the second-stage juvenile (J2) nematodes, the invasive stage of the nematode life cycle. This stage is optimal for isolating *M. incognita* proteins because it minimizes contamination from plant proteins and allows for easy and abundant collection of biological samples suitable for dCas9-based CAPTURE experiments. For consistency and reliability, we standardized our experiments using a defined number of nematodes, specifically 30,000 J2 (or eggs, for comparison).

**Fig. 2:**
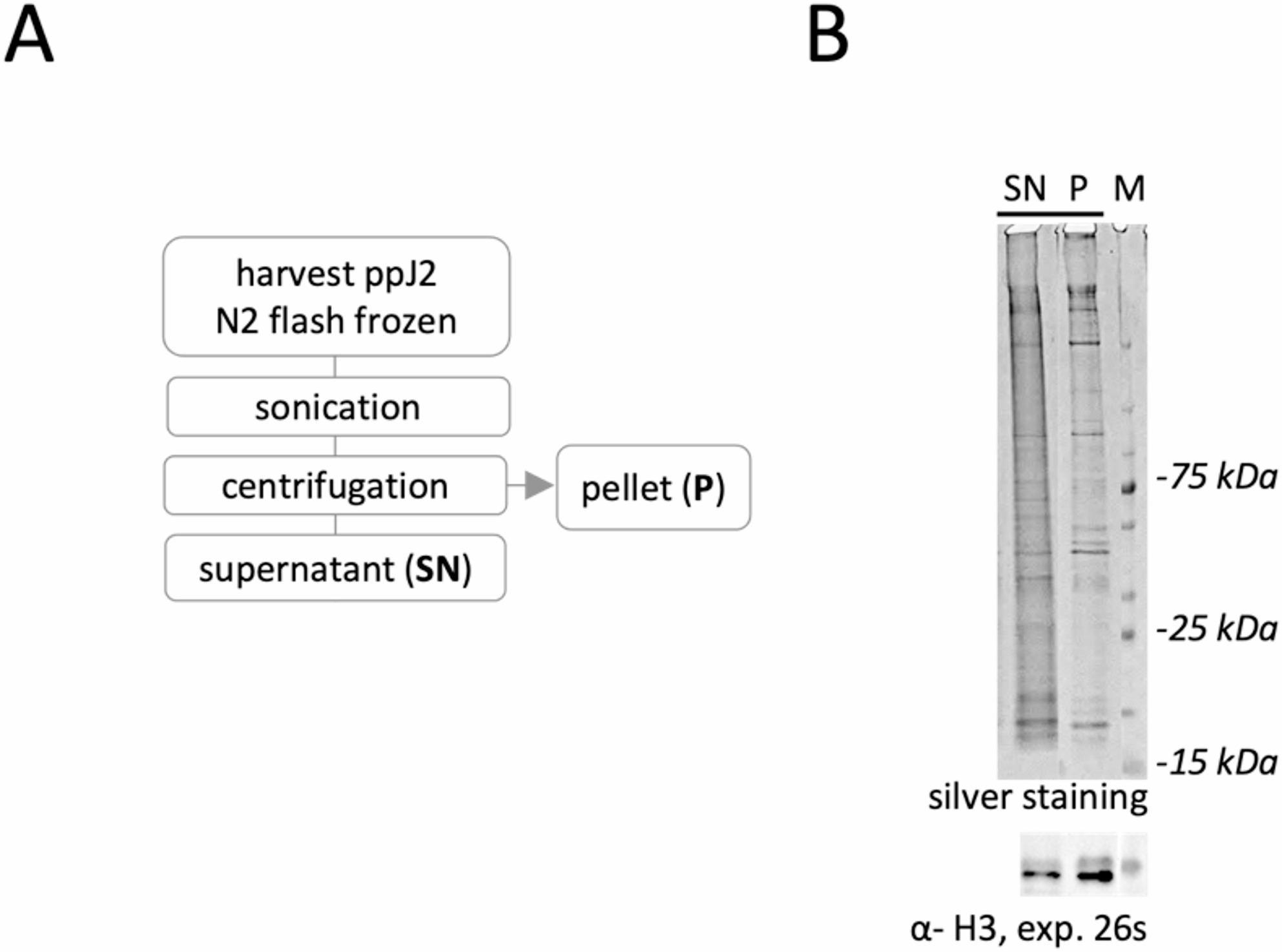
Quick and reliable method for solubilizing chromatin-associated proteins from *Meloidogyne incognita* J2. **A** Workflow illustrating the shearing and solubilization of chromatin-associated proteins using the Diagenode Bioruptor^®^ sonication system. **B** A batch of 30,000 J2 was resuspended in shearing buffer (iS1 buffer supplemented with a 1× protease inhibitor cocktail), subjected to sonication, and centrifuged. Supernatant (SN) and pellet (P) fractions were collected, separated by SDS–PAGE (4–15%), and visualized by silver staining (upper panel). Each lane contains protein obtained by the preparation of 500 ppJ2. The presence of Histone 3 (H3, ~ 16 kDa) was confirmed by immunoblotting using an anti-H3 antibody to assess chromatin enrichment in the different fractions (lower panel). Exposure time for chemiluminescent detection is indicated in seconds (s)

### Towards improved isolation of chromatin-associated proteins: sonication as a standalone method

Typically, ChIP workflows involve several key steps: tissue disruption, crosslinking, cell lysis, shearing of chromatin, and immunoprecipitation. In our preliminary optimization tests, we first evaluated conventional manual homogenizers commonly used, e.g. Dounce and Balch homogenizers. Although these approaches are standard for cellular and tissue homogenization, they proved largely unsuitable for breaking *M. incognita* J2, likely due to the nematode’s complex internal structure and highly resistant cuticle. To improve disruption efficiency, we subsequently applied a two-step strategy combining manual homogenization with ultrasonic treatment using the Bioruptor system. Sonication of chemically lysed J2 extracts slightly enhanced protein solubilization and visibly increased nematode breakage, as confirmed by SDS-PAGE. However, chromatin recovery remained incomplete, with limited detection of the chromatin marker Histone 3 (H3, ~ 16 kDa) in the soluble fraction and no detectable fragmented DNA, indicating that further optimization was needed. Detailed procedures and comparative analyses are provided in the Supplementary data section.

Given the limited DNA recovery and the labour-intensive nature of manual homogenization, we next assessed whether ultrasonic sonication alone could serve as an efficient and streamlined alternative. We established an optimized sonication-based workflow serving as an “all-in-one shearing system” capable of both J2 disruption and solubilization of chromatin proteome. In this approach, frozen batches of ∼ 30,000 J2 nematodes were sonicated under controlled conditions (duration, number of cycles, and cooling intervals). Standard sonication parameters used for cell suspensions (10 cycles, 30s ON/ 30s OFF) were ineffective for whole nematodes; therefore, we optimized the cycle duration to achieve efficient J2 disruption. After visual inspection, optimal settings consisted of 10 cycles of 90s ON/15s OFF, followed by 10 additional cycles of 30 s ON/ 15s OFF resulted in effective J2 disintegration into small clumps of cellular debris (Supplementary data section, Fig. 2). The prolonged ON time ensured efficient nematode disruption, while shorter OFF intervals minimized sedimentation of nematode debris between sonication cycles.

To evaluate the efficiency of this optimized sonication protocol, we compared protein profiles between soluble (SN) and insoluble (P) fractions obtained after centrifugation. Silver-stained SDS-PAGE revealed abundant protein content in (SN) enriched in nuclear and chromatin-associated proteins, while (P) contained residual proteins tightly bound to cellular debris. Immunoblotting using the chromatin H3 marker (H3, ~ 16 kDa) confirmed substantial enrichment of chromatin proteins in (SN). Although H3 remained more abundant in (P) than (SN) fractions, the solubilized H3 proportion in (SN) was markedly higher than two-step approach demonstrating improved chromatin recovery using sonication alone (Supplementary data section; (Fig. 2B)). A similar pattern was obtained using eggs as the starting material confirming the robustness of this method across developmental stages (Supplementary Fig. S1). Overall, this streamlined “all-in-one” sonication protocol provides an efficient and scalable solution for nematode disruption and chromatin protein solubilization, combining speed, reproducibility, and high sample throughput.

**Fig. 3:**
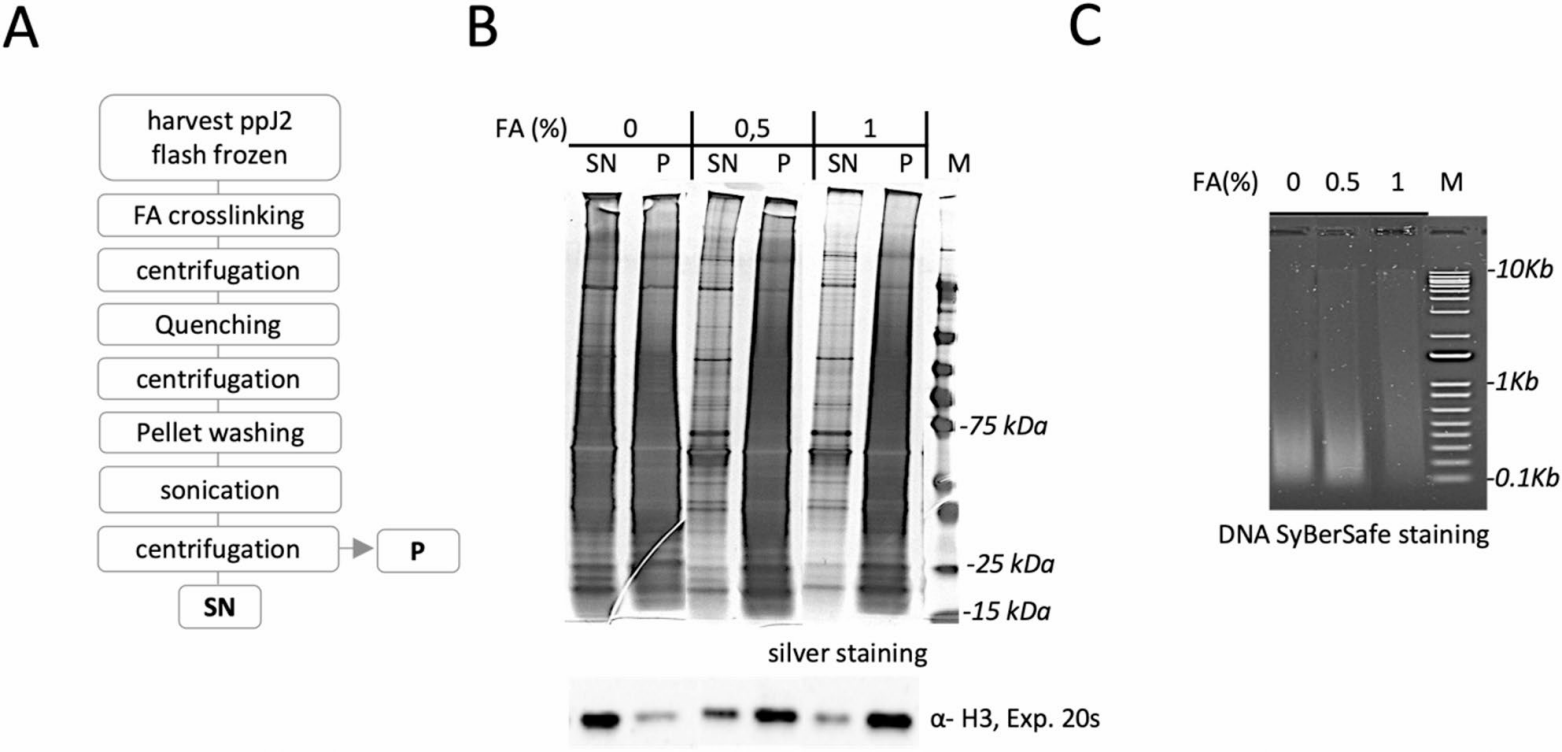
Influence of crosslinking conditions on chromatin protein solubilization and DNA shearing. **A** Pipeline for chromatin preparation following crosslinking of J2, utilizing the Diagenode Bioruptor^®^ as the sonication device. A batch of 30,000 J2 was were crosslinked using formaldehyde (FA), then centrifuged before formaldehyde quenching. Samples were subsequently centrifuged again, and pellets were washed with a physiological buffer. J2 were directly sonicated, followed by centrifugation, and their sheared chromatin profile was analyzed using silver-stained SDS-PAGE and agarose gel electrophoresis. **B** Aliquots of protein extracts, comprising supernatant (SN) and pellet (P) fractions, were collected, separated by SDS-PAGE (4–15%), then visualized by silver staining (upper panel). Each lane contains protein obtained by the preparation of 500 ppJ2.The presence of histone 3 (H3, ~ 16 kDa) was confirmed by immunoblotting using an anti-H3 antibody to evaluate the enrichment of chromatin among the fractions (lower panel). The exposure time for chemiluminescence is indicated in seconds (s). **C** SN samples generated in (**B**) were analyzed by SYBRSafe DNA electrophoresis, by loading 250 ng per lane in 2% agarose gel. Fixation at 0.5% formaldehyde presents optimal sheared chromatin, where most DNA fragments fall within the size range of 100–400 bp

### Crosslinking prior sonication for preserving DNA-protein interactions and isolating fragmented DNA in appropriate yield

Because the mechanical stress generated by sonication can disturb native protein-DNA interactions, including those involving transcription factors, we implemented an in situ crosslinking step prior to the “all-in-one shearing system”. Formaldehyde, a fast-acting conventional crosslinker, was used to stabilize protein-DNA interactions within *M. incognita* J2 before sonication. Since excessive crosslinking (high formaldehyde concentration and/or longer exposure) can promote nonspecific macromolecule associations and hinder chromatin solubilization, we empirically optimized the formaldehyde conditions by comparing the electrophoretic profiles of protein and DNA extracts. In brief, batches of 30,000 J2 nematodes were incubated in freshly prepared formaldehyde at two concentrations (0.5 and 1%) and with controlled exposure time. After quenching with Tris buffer and extensive washing to remove residual fixative, the samples were processed using the optimized sonication settings. Soluble (SN) and pellet (P) fractions were analysed to assess the impact of crosslinking on chromatin protein solubilization. Silver-stained SDS-PAGE revealed that (SN) from both crosslinking reactions exhibited similar protein patterns, which were clearly distinct from the untreated control lacking formaldehyde (Fig. 3B). DNA electrophoresis of (SN) fractions showed that 0.5% formaldehyde treatment produce efficient chromatin shearing, generating DNA fragments predominantly ranging from 100 to 400 bp – comparable to the untreated sample – while maintaining protein solubility (Fig. 3B, C). Both untreated and 0.5% formaldehyde-treated samples yielded approximately 5 µg of DNA from 30,000 J2 nematodes, roughly twice the amount recovered from the 1% formaldehyde-treated sample. Interestingly, when nematode eggs were used, an apparent well-balanced crosslinking efficiency with minimal protein loss and a suitable DNA size distribution was achieved with 1% formaldehyde for 10 min (Supplementary Fig. S2).

**Fig. 4:**
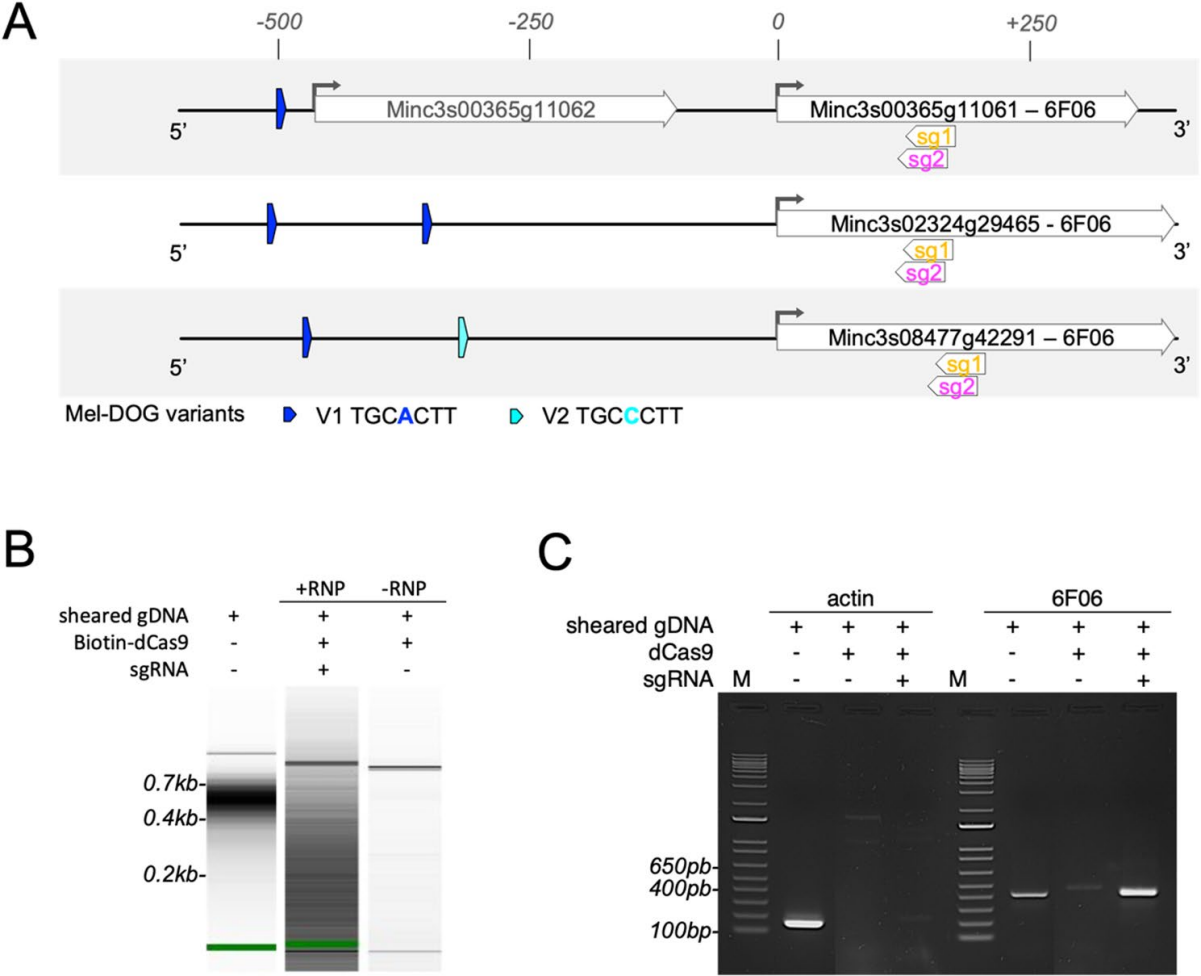
In vitro dCas9-based CAPTURE enrichment test using purified genomic DNA of *M. incognita* J2. **A** Map of the 6F06 effector gene loci targeted by in vitro dCas9-based CAPTURE. Three genomic regions of the 6F06 gene effector (Minc3s08477g42291, Minc3s02324g29465, Minc3s00365g11061) were used as genomic bait for in vitro dCas9-based CAPTURE assays. The promoters are characterized by the presence of a Mel-DOG box, existing in two variants (blue and turquoise arrows). The 6F06 promoter regions are targeted with two ribonucleoprotein (RNP) complexes corresponding to biotinylated dCas9:sgRNA1 and dCas9:sgRNA2. The selected sgRNAs (sg1 and sg2) are indicated. **B** Small fragments of genomic DNA from *M. incognita* J2 were generated by mechanical shearing using the bioruptor^®^, then incubated with a recombinant dCas9-Biotin loaded with the chosen guide RNAs (sg1 and sg2), or with no sgRNA as a negative control. RNP/gDNA mixtures were added to streptavidin-conjugated resin, followed by washing, and enriched gDNA fragments were pulled down using biotrap affinity purification technology. Enriched DNA fragments were subsequently analyzed using a Bioanalyzer. Gel images showed a broad distribution of DNA fragments targeted by the RNP dCas9:sgRNAs against 6F06 genomic regions compared to the negative control (without sgRNAs). **C** PCR analysis of the pulldown DNA fragments from (**B**) was performed targeting the 6F06 promoter regions of interest and Actin as negative control. DNA electrophoresis patterns from PCR demonstrated the presence of 6F06 in the enriched fraction compared to the negative control, while actin was poorly detected in this fraction of interest

### Choice of 6F06 effector as genomic region of interest and design of dCas9/sgRNA in a sequence specific manner

To demonstrate the proof-of-concept for in vitro dCas9-based CAPTURE in *M. incognita* J2, we focused on effector gene promoter regions containing the enriched motif known as the Mel-DOG box [1]. We established criteria for selecting the Mel-DOG genomic region as suitable bait based on previously published results, which indicated: (1) the presence of multiple Mel-DOG boxes within the first 1,000 bp upstream of known dorsal gland (DG) effector genes (considering both position and orientation), (2) Mel-DOG box associated to effector genes exhibiting the highest gene expression levels at J2 stage; and (3) the ability to design a specific guide RNA (sgRNA) targeting the DNA region of interest. Based on these criteria, we selected the promoter region of the *6F06* effector gene among 34 non redundant effector gene candidates [1]. Consistent with triploidy, three 6F06 homologues are present in the *M. incognita* genome (assembly version V3, 2017 [28]), referring to Minc3s00365g11061, Minc3s08477g42291 and Minc3s02324g29465. Each carries one Mel-DOG motif at − 497 bp or two Mel-DOG boxes from − 504/503 bp and − 347 bp relative to the start codon. The designed sgRNAs were chosen to target specifically a region conserved in the three 6F06 genomic loci, which may improve our enrichment analysis (Supplementary Table S1). The Mel-DOG motifs are positioned approximately 700 bp upstream of the designed sgRNA sequences (located 129 and 139 bp downstream of the start (Met) codon (Fig. 4A). The guide RNAs were designed to bind outside the 6F06 promoter region to avoid steric hindrance between dCas9 and promoter-associated proteins, for which we expected optimal DNA fragments sized around 800 bp (Fig. 4A) to ensure the isolation of related 6F06 promoter proteins of interest.

### *In vitro* Cas9/sgRNA cleavage against 6F06 PCR product: validation of the SgRNA choice

To validate the in vitro dCas9-based CAPTURE, we first performed an on-target Cas9 cleavage assay to confirm the specificity and efficiency of the sgRNAs targeting the *6F06* promoter region. The *6F06* PCR product (Minc3s02324g29465, 613 bp; Supplementary Fig. S3A, Supplementary Table S2) was successfully cleaved into two fragments using either sgRNA1 or sgRNA2 complexed with Cas9, generating fragments of 241/372 bp and 230/383 bp, respectively (Supplementary Fig. S3B).

### DNA fragments isolation in a sequence-specific manner by in vitro dCas9/sgRNA system, with the use of genomic

*M. incognita as input* To evaluate the effect of targeting dCas9 to the 6F06 locus, we next conducted the in vitro dCas9-based CAPTURE assay using purified genomic DNA. Sonication of the gDNA input sample (5 cycles of 30 s ON/ 30 s OFF) resulted in DNA size fragment distribution ranging from 400 to 700 bp, as assessed by Bioanalyzer (Fig. 4B). Fragmented *M. incognita* gDNA was incubated with either the biotin-tagged dCas9 / sgRNA complex, and on-target DNA fragments were isolated using a high-affinity Biotrap assay. To assess potential off-target binding by dCas9, a negative control lacking sgRNA was processed in parallel. The dCas9-sgRNA fraction of interest showed significant enrichment and a distinct distribution of dCas9-based CAPTURE DNA products compared to control samples, which included non-targeting dCas9 (without sgRNA) and the input sample. Target DNA regions were analysed by PCR amplification using primers specific for the 6F06 genomic region. A gene encoding actin was used as a negative control (Fig. 4C; Supplementary Table S2). The *6F06* PCR product was clearly found to be enriched in the + RNP sample compared to the – RNP sample. In contrast, a faint actin signal was detected in the + RNP sample, and was markedly lower than in the input DNA sample, indicating minimal off-target capture. Overall, these results validate the specificity and feasibility of using the in vitro dCas9-base capture assay to pulldown a defined genomic locus in *M. incognita*.

**Fig. 5:**
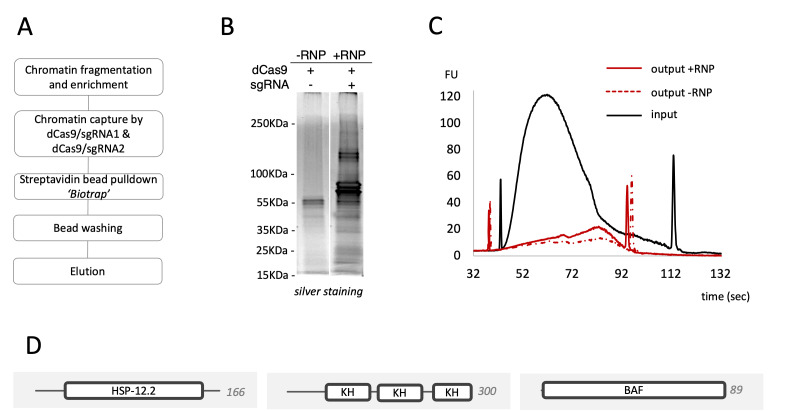
in vitro dCas9-based CAPTURE assay under native conditions. **A** Overview of the in vitro dCas9-based CAPTURE assay using *M. incognita* J2. Frozen J2 were crosslinked, and small chromatin fragments were obtained by mechanical shearing using the Bioruptor sonicator PLUS ; The recombinant dCas9-sgRNA complex (+ RNP, mixture with equal amounts of sgRNA1 and sgRNA2) were then added to the chromatin mixture, while recombinant dCas9 without sgRNAs (-RNP) served as negative control. Streptavidin-conjugated resin was added to the RNP/chromatin mixture and after washing, enriched chromatin was eluted using Biotrap technology. **B** Visualization of elution fractions from the biotrap assay on SDS-PAGE silver-stained gel electrophoresis, showing RNP targeting 6F06 loci (+ RNP) and the negative RNP control (-RNP). Several proteins were purified from the eluted fraction of interest. **C** DNA size distributions from eluted + RNP and – RNP fractions were analysed by Bioanalyzer. Sheared and crosslinked chromatin DNA fragments ranged from 200 to 600 bp (Input), while ChIP targeting 6F06 genomic regions or the negative control resulted in fragments from 500 to 800 bp (Output). A slight accumulation of DNA fragments was observed in the eluted fraction of interest. **D** Interproscan representation of selected proteins identified by mass spectrometry. In silico analysis revealed four candidates were potentially involved in chromatin regulation and transcription mechanisms associated with the expression of the 6F06 effector gene during the pre-parasitic stage of *M. incognita*. HSP-12.2 (A0A914L972; SHSP domain-containing protein) BAF (A0A914NCR7; Barrier to autointegration factor; KH (A0A914KPE9; K Homology domain-containing protein). Amino acid length is mentioned on the right side

### Isolation of chromatin complexes in a sequence-specific manner by in vitro dCas9/sgRNA system

We then scaled up the in vitro dCas9-based CAPTURE assay using chromatin of *M. incognita* J2 in a native cellular context (Fig. 5A). Protein and DNA patterns were analysed from immunoprecipitated fractions treated with dCas9-sgRNA targeting 6F06 genomic loci or non-targeting dCas9. As shown in Fig. 5B, the protein content captured by the in vitro dCas9-based CAPTURE was significantly enriched when sgRNAs were used compared to the negative control with no sgRNA. Similarly, chromatin fragments (referred to “output + RNP”) collected from the immunoprecipitated targeting sample exhibited distinct enrichment, with peak sizes around 500–800 bp, compared to the input sample (200–600 bp) (Fig. 5C). To identify 6F06 promoter-associated proteins, purified proteins were analysed by MS-based proteomics. Among the 193 proteins identified across the analysed samples, 17 proteins were found to be enriched in the + RNP sample compared to the control one (Supplementary Table S3). Among them, five are predicted to localize in the nucleus. To refine our candidate list, we prioritized proteins predicted to be associated with DNA, identifying three putative DNA-binding proteins: a small heat shock protein domain-containing protein (Minc10931a; nuclear, GO:0005634), a K homology domain-containing protein (Minc3s00047g02560; nuclear, GO:0005634; involved in gene expression regulation, GO:0010468), and the Barrier to autointegration factor (Minc16976; BANF1, DNA-binding, GO:0003677) (Fig. 5D; Supplementary Table S4). These results suggest that the in vitro dCas9-based CAPTURE assay allow the identification of potential protein partners associated with the 6F06 promoter region from *M. incognita* J2.

**Fig. 6:**
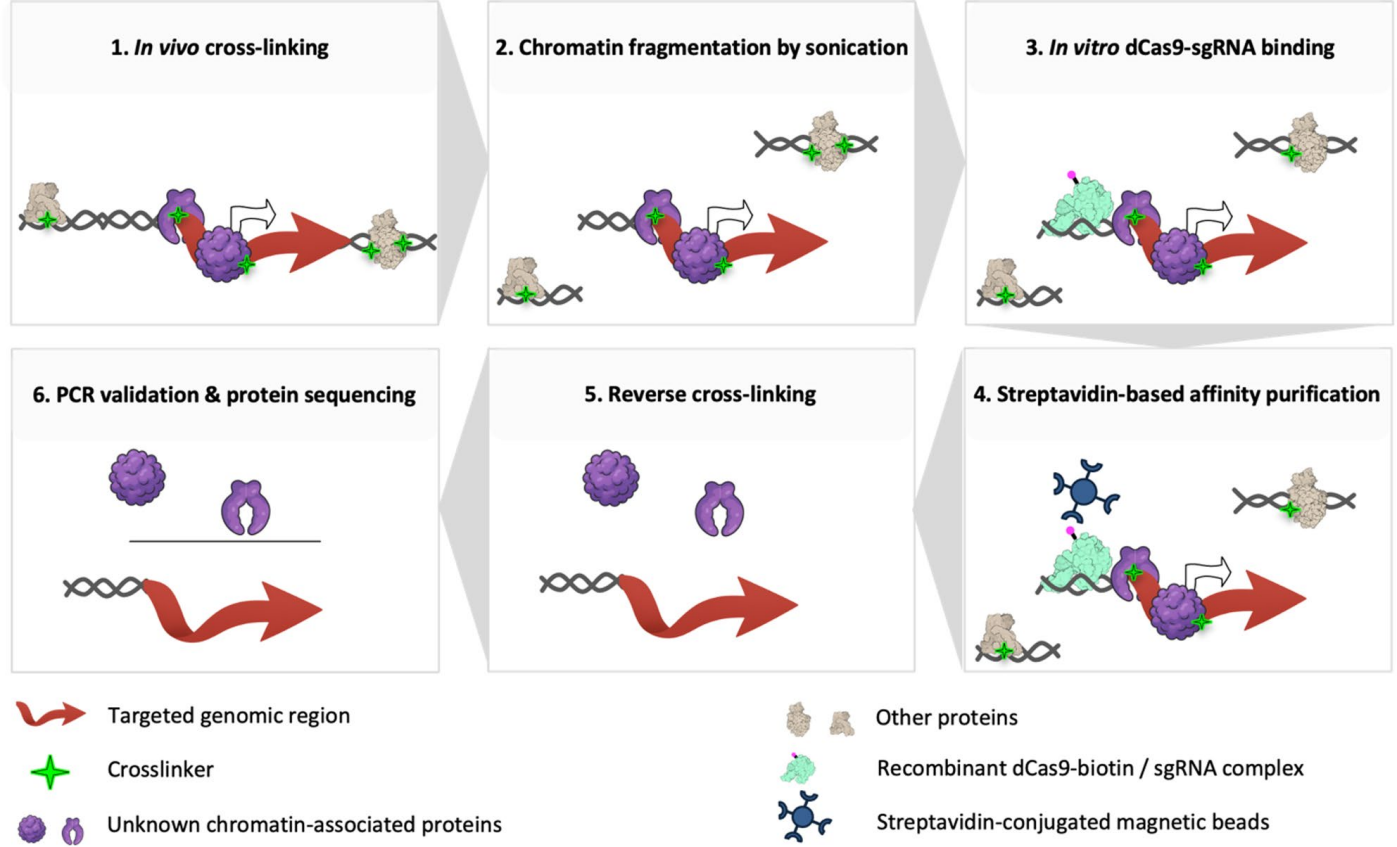
Workflow of the in vitro dCas9-based CAPTURE strategy applied to the phytonematode *M. incognita* J2. Whole J2 nematodes are crosslinked with formaldehyde to preserve protein-DNA interactions. Chromatin preparation involves homogenization and simultaneous chromatin fragmentation using a Bioruptor device. The sheared chromatin is recovered by centrifugation. The dCas9-sgRNA complex is used to target and bind the genomic region of interest. Chromatin-associated proteins are enriched through streptavidin bead capture, followed by reverse crosslinking. Interacting proteins are released, digested with trypsin, and analyzed by mass spectrometry for downstream data processing

## Discussion

### Developing a workflow to study chromatin-associated proteome in plant-parasitic nematodes

This study aimed to establish a robust workflow for chromatin isolation and preparation in *M. incognita* J2, paving the way for implementing the in vitro dCas9-based CAPTURE assay. Several optimizations were carried out (Fig. 6): (1) efficient J2 disruption and chromatin extraction, (2) optimized chromatin fragmentation, (3) adjustment of crosslinking conditions, (4) refinement of chromatin capture, and (5) improvement of the signal-to-noise ratio. Starting from 30,000 J2s, we obtained up to 5 µg of fragmented chromatin, demonstrating the feasibility of this workflow for studying chromatin dynamics in non-transformable nematodes.

### Challenges and solutions for chromatin isolation in nematodes

Previous studies in the well-established model system *C. elegans* have provided key insights in optimization of chromatin preparation, but adapting these protocols to *M. incognita* comes with unique challenges due to their small size, robust cuticle, and low chromatin content per unit of body weight [15, 29–31]. We evaluated two homogenization methods, e.g. Balch and Dounce homogenizers. While Balch homogenization has been successfully applied in *C. elegans*, it proved less compatible with the smaller nematode *M. incognita* [30, 32]. Manual dissociation, although feasible, required large quantities of nematodes due to sample loss, which impacted reproducibility, especially with low-input material. Additionally, higher densities of nematodes increased the mechanical effort needed, making the process time-consuming and raising the risk of destabilizing chromatin-protein complexes. We thus adopted sonication as an “all-in-one” method for both J2 disruption and chromatin shearing. Sonication conditions were optimized to both break down nematode tissues and fragment chromatin to sizes compatible with downstream dCas9-based CAPTURE assays. Several parameters were critical to efficient and uniform sonication, including preventing nematode sedimentation, which was more pronounced during the early stages of sonication when tissue disruption was minimal. Further optimal conditions of chromatin preparation may be necessary, such as adjusting fine-grained cycle intervals to ensure resuspension of the nematodes between rounds of sonication, and the sonication time and number of cycles or the Dounce homogenization protocol to improve chromatin release. Combining sonication with benzonase nuclease digestion proved beneficial for minimizing the destabilization of protein complexes [33].

### Preserving DNA–protein interactions through crosslinking

To maintain native chromatin integrity during sonication, we implemented an in situ formaldehyde crosslinking step. Crosslinking with 0.5% formaldehyde provided best compromise – preserving and yielding fragmented DNA (below to 1 kb) comparable to untreated samples, while maintaining protein solubility. Higher concentrations (1%) decreased DNA recovery but proved more effective for egg stage, indicating that optimal fixation varies across developmental stages. To calibrate crosslinking and sonication effects, we monitored H3 as a chromatin marker. Owing to its high degree of conservation and reliable commercial antibodies, H3 served as a robust indicator of chromatin enrichment and solubilization. Despite stringent sonication conditions, nucleosome interactions with genomic DNA remained intact, confirming the structural resilience of chromatin [34]. We also tested DNA polymerase II (PolII) to assess chromatin complex stability, but it yielded inconsistent signals [35]. Western blot analyses revealed reduced H3 levels in chromatin fractions following formaldehyde treatment, likely reflecting incomplete lysis or shearing caused by over-fixation. These results underscore the need to balance fixation strength to prevent artefactual crosslinking while ensuring efficient shearing. Importantly, an our data confirm that the use mild crosslinking enhances chromatin stability without impairing dCas9:sgRNA to anchor on chromatin. Thus, both sonication and formaldehyde crosslinking require careful, sample-specific optimization to produce high-quality chromatin suitable for dCas9-CAPTURE assay – overcoming challenges linked to low input material and tissue heterogeneity in nematodes [35, 36].

### Perspectives for chromatin studies in non-transformable nematodes

Conventional ChIP remains a powerful method for studying chromatin organization and transcriptional regulation, but its application to PPN is constrained by several technical barriers, notably antibody dependency and low chromatin input. The production of high-quality antibodies is particularly challenging, and ChIP further requires prior structural and protein-protein interaction knowledges of the target protein, information often lacking for nematode transcriptional regulators. The antibody-recognition epitope may be sterically masked within multiprotein complexes, preventing efficient immunoprecipitation. In contrast, dCas9-CAPTURE directly targets the genomic locus/loci, and the exceptionally strong biotin–streptavidin interaction ensures robust, high-affinity purification of associated chromatin complexes, independent of protein accessibility or local chromatin conformation. Nevertheless, steric hindrance can also be an issue with dCas9-CAPTURE, as the sgRNA target sequence itself may be masked or inaccessible. This highlights the importance of testing multiple sgRNAs and performing quality control by targeted PCR on the locus of interest. Other considerations about chromatin heterogeneity and low yield can limit enrichment specificity. Future improvements—such as sgRNA pre-screening, increased sequencing depth, or coupling CAPTURE with complementary techniques (e.g., CUT&Tag or proximity labelling)—should enhance detection sensitivity and broaden the applicability of this approach to other non-transformable nematode systems.

### Proof of concept: identifying chromatin-associated proteins at the 6F06 effector locus

A significant challenge remains in identifying parasitism gene expression regulators in *M. incognita*. In our study, the promoter region of the 6F06 effector gene was selected as a DNA bait to conduct dCas9-based CAPTURE, which identified candidates carrying a DNA-binding protein domain. However, these promising candidates associated with promoter regions of interest, remain to be experimentally characterized. From our dCas9-based CAPTURE experiments, we shortlisted three candidate proteins targeting 6F06 genomic loci. These included Barrier-to-autointegration (BANF1, BAF1) and two other proteins related to transcriptional regulation, a small heat shock protein (SHSP) domain-containing protein and K homology (KH) domain-containing protein. Although limited data are available on the role of BANF1 in parasitism, it has been shown to interact with SWI/SNF complexes [37]. BANF-1 can act as an epigenetic mediator, either activating or repressing gene expression [38]. Although we did not experimentally verify the role of these proteins in parasitism, several studies suggest a potential role for SWI/SNF complexes in the regulation of parasitism and stress responses [39, 40]. These findings mirror with the previous work in *M. incognita*, which highlighted that approximately 20% of effector genes are regulated by histone modification dynamics. For instance, 6F06 is one of them that exhibits repressive expression during the transition from eggs to juvenile stages (J2), and this repression is associated with H3K9me3 mark [14]. Overall, it would be valuable to further study their functional roles to evaluate their significance in parasitism at the early stages of infection.

The dCas9-based CAPTURE system not only captures chromatin but can also isolate locus-specific long-range DNAs or chromatin-associated RNAs, such as long non-coding RNAs (lncRNAs), which are known to act as trans-regulatory elements in concert with regulatory proteins to control gene expression [41]. Evidence from the literature shows that SWI/SNF complexes require guidance from transcriptional regulators, histone modifiers, or lncRNAs to interact effectively with target loci, due to their loss of DNA binding specificity and low intracellular concentrations [40, 42]. This underscores the value of using dCas9-based CAPTURE, as illustrated by [40], which identified interacting proteins, RNAs and DNAs, ultimately providing insights into parasitism gene regulation models. Remarkably, regarding effector loci, sgRNAs could be designed to target specific groups of effectors, provided that conserved sequences exist among their promoters. Beyond candidate identification, the methodology offers the potential to unravel the common signalling networks controlling effector gene expression.

### Conclusion

We developed and optimized a scalable chromatin isolation and dCas9-based CAPTURE workflow tailored for *M. incognita* J2. This represents a methodological breakthrough for exploring DNA–protein interactions in non-transformable plant-parasitic nematodes. Identification of chromatin regulators such as BANF1 underscores the involvement of epigenetic mechanisms in parasitism gene regulation. The method can be extended to other effector loci or gene families using customized sgRNAs, providing a valuable framework for dissecting the regulatory networks underlying nematode parasitism.

## Materials and methods

### Nematode collection

*Meloidogyne incognita* race Morelos acquired from Laboratory of Nematology of Wageningen University & Research was used in all experiments. The nematode culture and maintenance were conducted on greenhouse tomato roots (*Solanum lycopersicum* cv. Roma) growing in a mixture of substrate/sand (ratio 2:1). Infected plants were watered daily and maintained approximately at 23 °C all year. *Meloidogyne incognita* eggs were collected every 8 to 11 weeks for reinoculation (depending on the period of the year). Nematode extraction and J2 hatching were performed as described in [43]. J2 were freshly collected every two days, left to settle at room temperature, and subsequently cleaned with 500 µL Hank’s Balanced Salt solution buffer (HBSS) (5 mM KCl, 0.4 mM KH_2_PO_4_, 4 mM NaHCO_3_, 50 mM NaCl, 0.3 mM Na_2_HPO_4_, and 5.5 mM Glucose) after centrifugation at 13,000 rpm for 1 min. After removing HBSS buffer, batches of 30,000 J2 (or eggs) were stored at -80 °C for several weeks, termed hereafter frozen J2, and used for all of the experiments.

### Workflow from the one-step chromatin Preparation to dCas9-based CAPTURE assay

#### J2 sample Preparation

Aliquots containing approximately 30,000 *M. incognita* J2 or eggs, previously washed in HBSS, centrifuged, and stored as pellets at − 80 °C until use, were thawed on ice and briefly equilibrated to room temperature. Unless otherwise stated, all buffers were freshly supplemented with a 1× protease inhibitor cocktail (Roche, #11873580001).

#### Crosslinking

Given that the kinetics and efficiency of formaldehyde-mediated crosslinking are temperature-dependent [44], reactions were performed at room temperature using freshly opened single-use methanol-free 16% formaldehyde (w/v) (Pierce™) ampules. Each batch of 30,000 J2 was resuspended in 500 µL of fixative buffer (0.5%, 1% formaldehyde, or formaldehyde-free control in HBSS) and gently rotated (21 rpm) to ensure homogeneous fixation. Crosslinking was quenched by adding 750 mM Tris-HCl (pH 8.0) and incubating for 5 min. under gentle rotation [45]. Samples were then maintained at 4 °C and washed twice with 500 µL of cold HBSS buffer to remove residual fixative. J2 pellets were collected by centrifugation (2,700 rpm, 5 min, 4 °C) and resuspended in 100 µL of cold shearing buffer (iS1 buffer, Diagenode Chromatin EasyShear kit - Low SDS (C01020013) followed by 10 min. of incubation under rotation (21 rpm, 4 °C) to equilibrate the sample prior to shearing.

#### Simultaneous disruption and chromatin shearing

Crosslinked or non-crosslinked J2 were disrupted and chromatin simultaneously sheared using the Bioruptor PLUS sonication system (Diagenode, #B01020004). Sonication was performed in 1.5 ml TPX microtubes (#M-50001, Diagenode) under high power, using the following optimized program: 10 cycles of 90 s ON / 15 s OFF, with gentle tube mixing after every 10 cycles, followed by 10 additional cycles of 30 s ON / 15 s OFF in a 4 °C circulating water bath (without floating ice). After sonication, samples were centrifuged at 16,000 x *g* for 10 min. at 4 °C to separate the soluble sheared chromatin fraction from debris, the resulting supernatant, containing sheared chromatin, was collected and aliquoted for subsequent analyses (e.g. DNA purification, SDS-PAGE, and Western blotting). The pellet, containing residual J2 or egg debris, was visually inspected under a light microscope to confirm the efficiency of nematode disruption. Chromatin samples were stored at -20 °C or -80 °C until further use.

#### Reverse crosslinking and DNA recovery

To evaluate the effect of sonication procedure on chromatin shearing, we conducted reverse DNA crosslinking and purification. As described in [45], 20 µL of sheared chromatin was de-cross-linked with the addition of 95 µl TE/SDS buffer (10 mM Tris pH8, 1 mM EDTA, 1% SDS) and incubated overnight at 65 °C in a thermoshaker (800 rpm). Samples were then treated with 2.5 µL RNAseA (10 mg/ml) for 30 min. at 37 °C followed by the addition of 20 µL proteinase K (10 µg/µl) and incubation at 55 °C for 2 h. Samples were then cleaned with MicroChIP DiaPure columns kit (#C03040001, Diagenode) and eluted in 8 µL nuclease free water, following the manufacturer’s instructions. Fragmented DNA samples were visualized on a 2% agarose gel by electrophoresis (loading 250 ng per lane and) and on an Agilent 2100 Bioanalyzer High Sensitivity DNA Chip (#G2938-68000) following the manufacturer’s recommendation.

#### Analysis of proteins by silver-stained SDS-PAGE and Western blotting

Pellet and all supernatant fractions were resuspended in Laemmli urea buffer (126 mM Tris-HCl pH 6.8, 8 M Urea, 20% Glycerol, 4% SDS, 0.02% bromophenol blue) respectively, then boiled at 80 °C for 8 min. Aliquots corresponding to approximatively 500 J2 were loaded per lane for SDS-PAGE analysis (Mini-PROTEAN TGX precast protein gels 4–15%, #4561086) and stained using the Pierce Silver Stain kit (Thermo Scientific™) following the manufacturer’s protocol. For immunoblot analysis, aliquots from each fraction were separated by SDS-PAGE (4 − 1% gradient) and transferred to PVDF membranes under the following conditions: 14 V/1.3 mM 7 min. on Bio-Rad instrument. Membranes were washed twice in Tris-buffered saline containing 01% Tween-20 (TBS-T; 50 mM Tris-Cl, pH 7.6, 150 mM NaCl) for 5 min. and then blocked overnight at 4 °C with5% (w/v) non-fat dry milk in TBS-T under gentle agitation. Blots were then incubated for 1 h at room temperature with a rabbit polyclonal primary antibody recognizing an invariant domain of Histone 3 (H3, abcam #ab1791, dilution 1:2500), followed by a 1 h incubation with HRP-conjugated goat anti-rabbit IgG secondary antibody (1:10,000 dilution) in3% milk-TBS-T buffer. Chemiluminescent signals were developed using the Clarity ™ Western ECL substrate (#1705062, Bio-Rad) for 3 min. and imaged with Bio-Rad ChemiDoc XRS + Gel Imaging System.

#### In vitro dCas9-based CAPTURE

Protocol for the in vitro dCas9-based CAPTURE assay was adapted from the experimental procedure realized in [25, 26, 46].

*(A) in vitro dCas9-based CAPTURE using genomic DNA as bait* The procedure of in vitro dCas9-based CAPTURE from gDNA of approximatively 50,000 *M. incognita* J2 were as follows:

*Genomic DNA extraction* J2 were ground in 50 µL lysis buffer (0.1 M Tris-HCl pH 8.0, 0.5 M NaCl, 50 mM EDTA, 1% SDS) using a micro- pestle for 30 s, followed by addition of 450 µL lysis buffer an 4 µL proteinase K (20 mg/ml). Samples were incubated for 24 h at 55 °C in a Thermomix. The J2 lysate was then treated with 10 µL RNAse A (10 mg/mL) for 30 min. at 37 °C.

*Genomic DNA precipitation* gDNA was precipitated by adding an equal volume of Na-Ac buffer (2.5 M Ammonium acetate, 0.3 M sodium acetate), followed by 0.7 volume of isopropanol at room temperature. The mixture was centrifuged at 15,000 x *g* for 30 min. at 4 °C. The pellet was washed with 70% ethanol at equal volume, centrifuged again (15, 000 x *g*, 15 min, 4 °C), air-dried for 10 min., and resuspended in 8 µL nuclease-free water for 2 h at 55 °C.

*Genomic DNA sonication and purification* Purified gDNA was diluted in 100µL of the cold shearing buffer (iS1, Diagenode Chromatin EasyShear kit - Low SDS #C01020013), and sonicated using the Diagenode Bioruptor PLUS (#B01020004) at high power for 5 cycles of 30 s ON/ 30 s OFF, at 4 °C without floating ice. The resulting DNA fragments averaged 400–700 bp in size. Sheared DNA was purified with the MicroChIP DiaPure columns kit (#C03040001, Diagenode) and eluted in 8 µL nuclease-free water (yield ∼ 6 µg (ratio A260/280 nm 1.85).

*Precleaning of magnetic beads* Thirty microliters of Pierce ™ Streptavidin magnetic beads magnetic beads (#PI88816, Thermo Scientific ™) were washed twice in 500 µL of cold Phosphate buffered saline (PBST, 1X PBS pH 7.4, 0.1% Tween-20) using a magnetic rack for 3 min. on ice, then resuspended in 500 µL of cold PBST-B buffer (completed with 0.1% BSA).

*Hybridizing dCas9-RNP complex to magnetic beads* The RNP dCas9-sgRNA complex was assembled at a 1:5 molar ratio for hybridization with 1 µg of sheared gDNA. A total of 1.56 µg recombinant dCas9 protein was incubated with pre-cleaned magnetic beads in the PBST-B buffer for 2–3 h on a rotating wheel (in cold chamber). Beads were collected with a magnetic rack, washed twice, and resuspended in 100 µL in vitro CRISPR buffer and placed on ice for 3 min. with the magnetic frame to discard the supernatant [36]. Next, the magnetic beads complex was washed twice and resuspended in 100 µl CRISPR buffer (20 mM HEPES pH 7.1, 150 mM KCl, 0.1 mM EDTA, 10 mM MgCl2, 0.5 mM DTT added freshly, and 1X EDTA-free protease inhibitor cocktail, Roche). Each sgRNA (0.85 µg) was then added and incubated with the bead-dCas9 complex for 10 min. at 37 °C.

*Hybridization of sheared genomic DNA and affinity isolation using the in vitro dCas9-CAPTURE system* One microgram of sheared gDNA was diluted 10-fold in CRISPR buffer to minimize SDS interference on dCas9 activity, then added to the pre-assembled dCas9-RNP-bead complex supplemented with RNase inhibitor (40 U of RNasin, Promega). The reaction was incubated for 30 min. at 37 °C with shaking (650 rpm, Thermomixer). gDNA/RNP-bead mix were washed sequentially using the magnetic rack: four times with 1 mL of cold low-salt buffer (20 mM Tris-HCl pH 8.0, 150 mM NaCl, 2 mM EDTA, 0.1% Triton X-100, 0.03% SDS supplemented with 5U of RNAsin (invitrogen) and 1X protease inhibitors cocktail), once with 1 mL of cold TBS-NP40 (50 mM Tris-HCl pH 7.5, 150 mM NaCl, 0.5% NP-40), and three times with 1 mL of cold TE buffer (10 mM Tris-HCl pH7.5, 1 mM EDTA). Bound complexes were eluted in 100 µL SDS-ChIP buffer (20 mM Tris-HCl pH 8.0, 2 mM EDTA, 150 mM NaCl, 0.1% SDS, 1% Triton X-100 supplemented with 1X Protease inhibitor cocktail) and incubated overnight at 70 °C in a thermocycler. The following day, samples were cooled to room temperature, mixed with 100 µL of TE buffer, and the captured gDNA fragments were then purified with MicroChIP DiaPure columns kit (#C03040001, Diagenode). Purified gDNA fragments were analysed to assess hybridization efficiency. The MiActin gene promoter served as an internal reference. The truncated 6F06 promoter was PCR-amplified using primers listed in Supplementary Table S2, with DNA samples incubated with or without the dCas9-RNP complex as templates.

*(B) in vitro dCas9-based CAPTURE using native chromatin as bait* Preparation of biological material, crosslinking and sonication were performed as described above. Crosslinking and sonication parameters are specified in the corresponding figure legends. Each test sample contained 5 µg of crosslinked and sheared chromatin, prepared from 30,000 J2. After centrifugation, the input fragmented chromatin (peaking at 200–600 bp) was immunoprecipitated with the dCas9-biotin system as described above. Captured fractions were subsequently analysed by Bioanalyzer, mass spectrometry, SDS-PAGE.

*MS-based proteomic analyses* Proteins were solubilized in Laemmli buffer and stacked in the top of a 4–12% NuPAGE gel (Invitrogen), stained with Coomassie blue R-250 (Bio-Rad) before in-gel digestion using modified trypsin (Promega, sequencing grade) as previously described [47]. The resulting peptides were analysed by online nanoliquid chromatography coupled to MS/MS (Ultimate 3000 RSLCnano and Orbitrap Exploris, Thermo Fisher Scientific) using a 60-min. gradient. For this purpose, the peptides were sampled on a precolumn (300 μm x 5 mm PepMap C18, Thermo Scientific) and separated in a 75 μm x 250 mm C18 column (Aurora Generation 3, 1.7 μm, IonOpticks). The MS and MS/MS data were acquired using Xcalibur (Thermo Fisher Scientific).

Peptides and proteins were identified by Mascot (version 2.8.0, Matrix Science) through concomitant searches against homemade databases containing the predicted protein sequences of *M. incognita* and of classical contaminant proteins found in proteomic analyses (keratins, trypsin.). Trypsin/P was chosen as the enzyme and two missed cleavages were allowed. Precursor and fragment mass error tolerances were set at respectively at 10 and 20 ppm. Peptide modifications allowed during the search were: Carbamidomethyl (C, fixed), Acetyl (Protein N-term, variable) and Oxidation (M, variable). The Proline software [48] (version 2.3.3) was used for the compilation, grouping, and filtering of the results (conservation of rank 1 peptides, peptide length ≥ 6 amino acids, false discovery rate of peptide-spectrum-match identifications < 1% [49], and minimum of one specific peptide per identified protein group). Proline was then used to perform a weighted spectral counting (WSC)-based comparison of the samples. Proteins were considered enriched in a sample compared to another if they were identified by at least 3 WSC and identified only in one sample or enriched at least 5 times in a sample on the basis of WSC.

*Cas9 digestion assay* To assess the efficiency and specificity of the designed sgRNAs, an in vitro DNA cleavage assay was conducted using PCR-amplified fragments of 6F06 gene locus (Minc3s08477g42291). The target region was amplified from *M. incognita* genomic DNA using specific primers (Supplementary Table S2) and the Phusion High-Fidelity PCR kit (New England Biolabs). PCR products were analysed by agarose gel electrophoresis, then purified with the Monarch DNA purification kit. The Cas9 cleavage reaction was assembled in thermocycler as follows: 260 nM sgRNA and 260 nM Cas9 protein (CAS9PROT, Sigma) were pre-incubated in 1X NEB3.1 buffer for 10 min. at 25 °C to allow RNP formation, followed by the addition of 26 nM purified PCR product (final molar ratio 10:10:1). The reaction was incubated for 1 h at 37 °C and subsequently heat-inactivated at 80 °C for 20 min. Cleavage efficiency of the effector gene fragments was assessed by electrophoresis on a 2% agarose gel.

## Supplementary Information

Below is the link to the electronic supplementary material.

Supplementary Material 1.

Supplementary Material 2.

## Data Availability

No datasets were generated or analysed during the current study.
