# Supplementary material for: Profiling DNA-protein interactions in Meloidogyne incognita using dCas9-based affinity purification
Source: Plant Methods. 2026 Jan 4;22:20. doi: 10.1186/s13007-025-01475-5 (PMC12895963; doi:10.1186/s13007-025-01475-5)
Supplement: Supplementary file 1 — Supplementary Material 1. [file 13007_2025_1475_MOESM1_ESM.docx]

Profiling DNA-protein interactions in *Meloidogyne incognita* using dCas9-based affinity purification

**Authors**

Caroline Bournaud^1*^, Alwéna Tollec^2^, Etienne G J Danchin^3^, Yohann Couté^2^ and Sebastian Eves-van den Akker^4*^

**Affiliations :**

^1^ Microbiologie, Adaptation et Pathogénie, UMR5240, Univ Lyon, Université Lyon 1, Bayer SAS, 69622 Villeurbanne, France.

^2^ Univ. Grenoble Alpes, Inserm, CEA, UA13 BGE, CNRS, CEA, UAR2048 ProFI, 38000 Grenoble, France.

^3^ Institut Sophia Agrobiotech, INRAE, Université Côte d'Azur, CNRS, 400 routes des Chappes, 06903, Sophia-Antipolis, France

^4^ The Crop Science Centre, Department of Plant Sciences, University of Cambridge, Cambridge CB2 3EA, United Kingdom.

***Correspondence:**

Caroline Bournaud

caroline.bournaud@univ-lyon1.fr

Sebastian Eves-van den Akker

[se389@cam.ac.uk](mailto:se389@cam.ac.uk)

# **Supplementary figures**

**
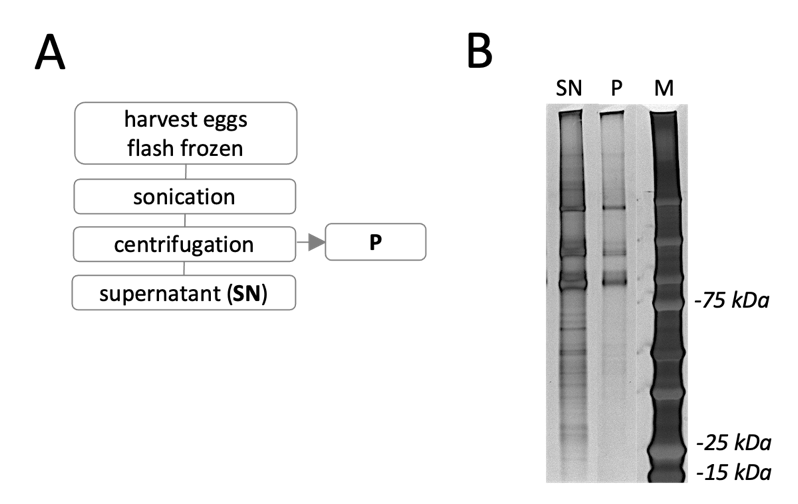
**

**Supplementary Figure S1. Protein extracts after homogenization and sonication steps from *M. incognita* eggs. (A)** Pipeline for chromatin isolation and shearing process using the Diagenode Bioruptor® as the sonication device. **(B)** Frozen eggs were directly sonicated, followed by centrifugation. Aliquots of protein extracts, comprising supernatant (SN) and pellet (P) fractions, were collected and separated by SDS-PAGE (4-15%), then visualized by silver staining. Each lane contains protein obtained by the preparation of 500 ppJ2. The presence of Histone 3 (H3, ~16 kDa) was not detected by immunoblotting using an anti-H3 antibody.


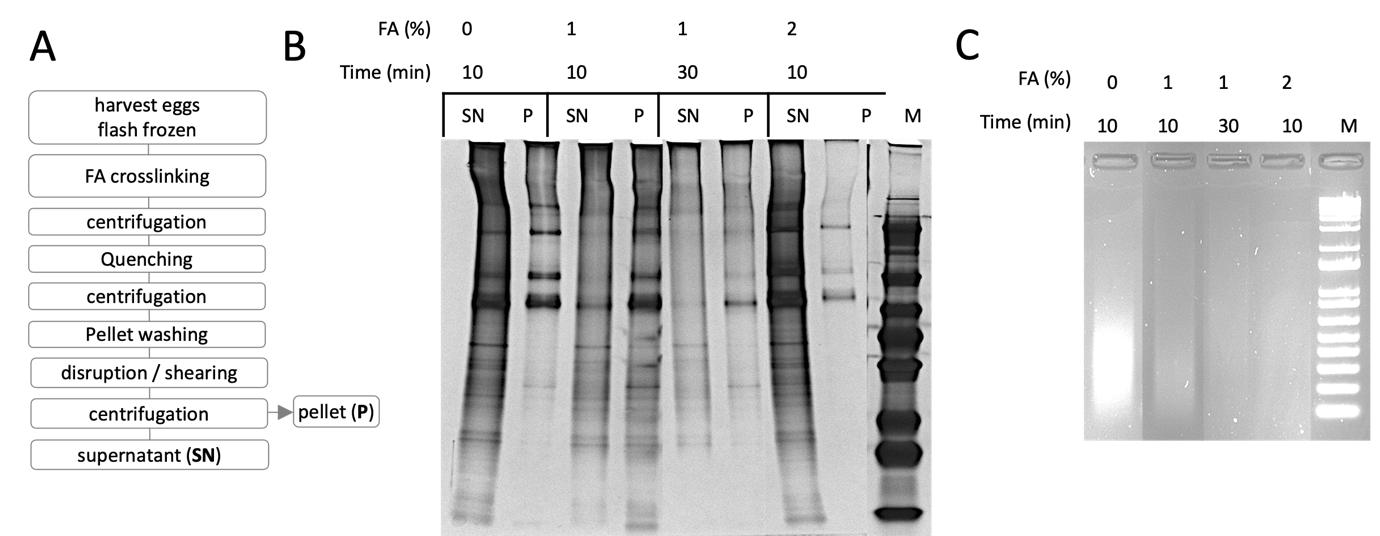


**Supplementary Figure S2. Effect of crosslinking conditions on chromatin-protein isolation and DNA shearing in *M. incognita* eggs.** **(A)** Pipeline for chromatin preparation following crosslinking of eggs, utilizing the Diagenode Bioruptor® as the sonication device. Frozen eggs were crosslinked using formaldehyde, then centrifuged before FA quenching (Tris-HCl, 5 min). Samples were subsequently centrifuged again, and pellets was washed with a physiological buffer. Crosslinked eggs were directly sonicated, followed by centrifugation, and their sheared chromatin profile was analyzed using silver-stained SDS-PAGE and agarose gel electrophoresis. **(B)** Aliquots of protein extracts, comprising supernatant (SN) and pellet (P) fractions, were collected, separated by SDS-PAGE (4-15 %), then visualized by silver staining. Each lane contains protein obtained by the preparation of 500 ppJ2. The presence of histones was not observed by immunoblotting with histone H3 antibody. **(C)** Chromatin samples generated in (B) were analyzed by SYBRSafe DNA electrophoresis in 2 % agarose gel. Fixation at 1 % formaldehyde for 10 min. presents optimal time and concentration for shearing chromatin, where most DNA fragments were distributed from 100 to 400 bp.

**
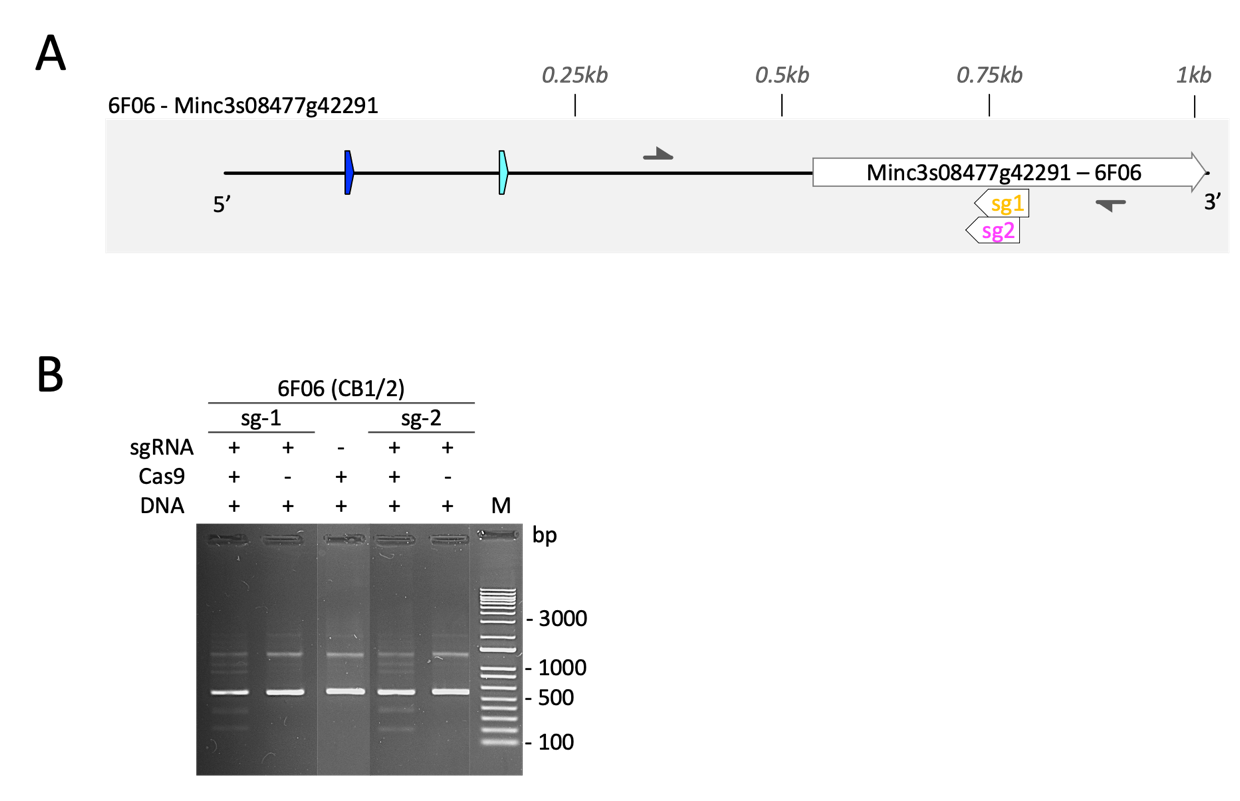
**

**Supplementary Figure S3. *in vitro* Cas9 assay to assess the cleavage efficiency from chosen sgRNAs targeting PCR-amplified 6F06 locus** (**A**) Map of the 6F06 effector genomic locus (Minc3s08477g42291), showing the regions targeted by the recombinant Cas9/sgRNA complexes. The promoter region contains Mel-DOG boxes, existing in two variants (blue and turquoise arrows). The 6F06 promoter region is targeted with two ribonucleoprotein (RNP) complexes, each composed of the active form of Cas9 and a specific sgRNA (sg1 and sg2), with their positions are indicated. PCR amplification was performed using flanking primers (grey arrows) to generate the DNA template for the assay (**B**) Representative agarose gel showing results from *in vitro* digestion assays testing the cleavage efficiency of two sgRNAs targeting the PCR-amplified 6F06 locus. Purified PCR matrices incubated with recombinant Cas9 protein lacking a specific sgRNA (-) showed no cleavage, while the addition of sgRNA/Cas9 complexes led to moderate cleavage of the target PCR product. M = 1 kb DNA ladder.
