# Supplementary material for: Profiling DNA-protein interactions in Meloidogyne incognita using dCas9-based affinity purification
Source: Plant Methods. 2026 Jan 4;22:20. doi: 10.1186/s13007-025-01475-5 (PMC12895963; doi:10.1186/s13007-025-01475-5)
Supplement: Supplementary file 2 — Supplementary Material 2. [file 13007_2025_1475_MOESM2_ESM.docx]

*Supplementary Data Section for*

**Running title**

Profiling DNA-protein interactions in *Meloidogyne incognita* using dCas9-based affinity purification

**Authors**

Caroline Bournaud^1*^, Alwéna Tollec^2^, Etienne G J Danchin^3^, Yohann Couté^2^ and Sebastian Eves-van den Akker^4*^

**Affiliations :**

^1^ Microbiologie, Adaptation et Pathogénie, UMR5240, Univ Lyon, Université Lyon 1, Bayer SAS, 69622 Villeurbanne, France.

^2^ Univ. Grenoble Alpes, Inserm, CEA, UA13 BGE, CNRS, CEA, UAR2048 ProFI, 38000 Grenoble, France.

^3^ Institut Sophia Agrobiotech, INRAE, Université Côte d'Azur, CNRS, 400 routes des Chappes, 06903, Sophia-Antipolis, France

^4^ The Crop Science Centre, Department of Plant Sciences, University of Cambridge, Cambridge CB2 3EA, United Kingdom.

***Correspondence:**

Caroline Bournaud

caroline.bournaud@univ-lyon1.fr

Sebastian Eves-van den Akker

[se389@cam.ac.uk](mailto:se389@cam.ac.uk)

**This word document includes:**

Supplemental result and discussion

Supplemental Figures and legends S1-S3

Supplemental material and methods

Supplemental references

**SUPPLEMENTAL RESULT AND DISCUSSION**

This supplementary file provides additional data supporting the development of the chromatin preparation workflow (‘all-in-one system’), including optimization of homogenization and chromatin recovery steps.

**
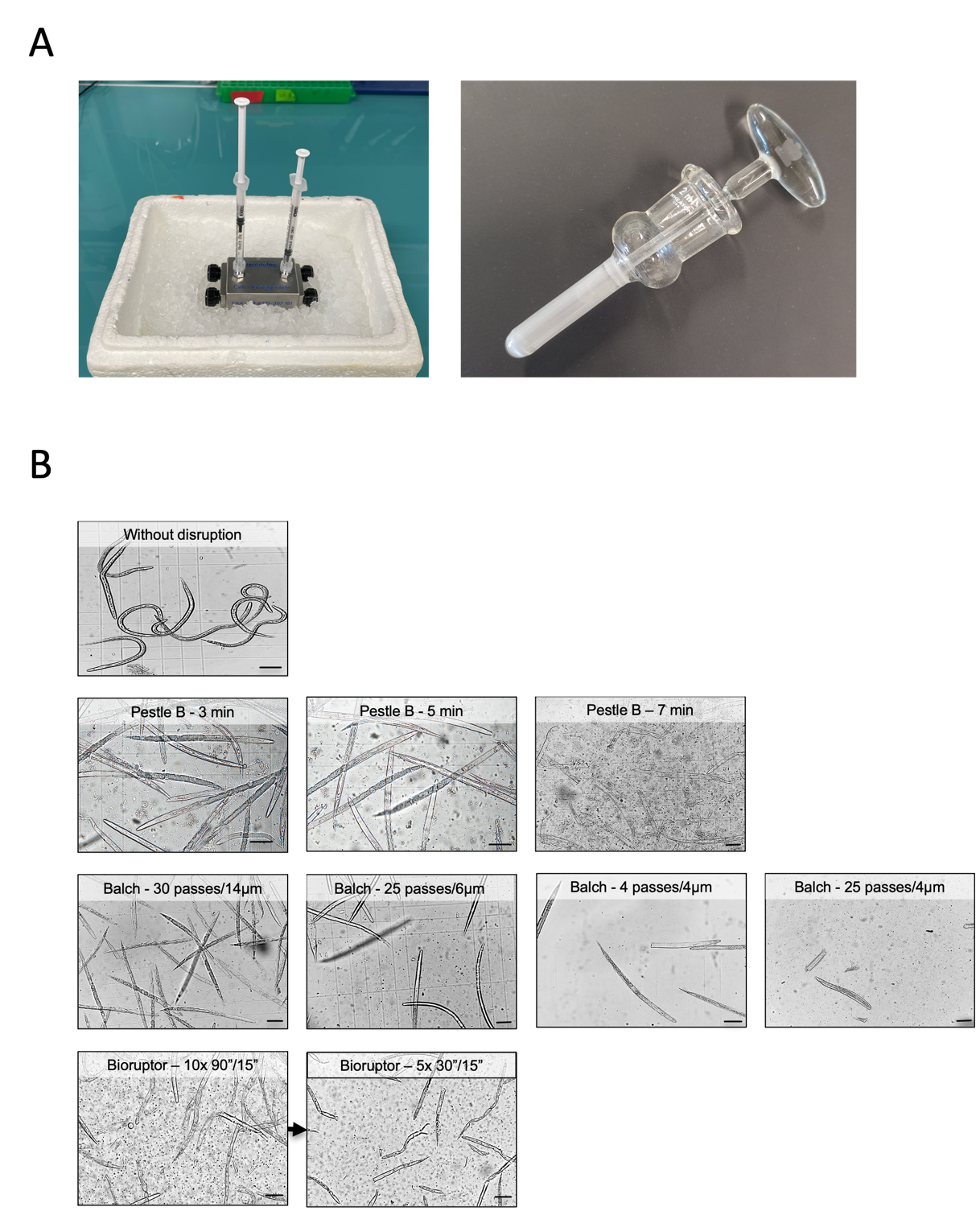
**

**Supplementary Figure S1.** Optimization of homogenization methods for the PPN *Meloidogyne incognita*. **(A)** Photos of the Balch homogenizer (left) and Dounce homogenizer (right). **(B)** Approximately 30,000 second-stage juveniles (J2) were resuspended in homemade Hank’s Balanced Salt solution (HBSS) buffer and gently disrupted on ice to generate a relative homogenate. Worm lysates were processed using various mechanical homogenization techniques: Dounce homogenizer with loose (A) or tight (B) pestle, Balch homogenizer using an 4- to 14 µm clearance ball bearing and with different combinations of syringe pass numbers, and Bioruptor device under different time and cycle conditions. All homogenization steps were performed on ice or at 4°C to maintain sample integrity. While these methods were effective in grinding nematode cells to subcellular components, none achieved complete homogenization. Further validation steps, including nuclear extract preparation, chromatin proteome analysis, and DNA isolation, were performed to ensure sample quality and compatibility for downstream analyses. Scale bar: 50µm

**Evaluation of homogenization techniques** – An essential step in any ChIP assay is the efficient homogenization of biological samples to release chromatin-associated proteins. This process is particularly challenging in plant-parasitic nematodes (PPN) due to their robust cuticle and complex tissue organization. To overcome this, we evaluated several mechanical disruption techniques previously reported for nematodes such as *C. elegans* [1-4]. Based on these precedents, we tested three homogenization devices: a Dounce homogenizer (pestles A and B with different clearance), a Balch homogenizer (metal chamber with defined ball bearings), and a 29-gauge syringe needle system. Following the gentle grinding on ice, microscopic examination revealed that the Dounce homogenizer (pestle B) efficiently disrupted M. incognita J2s, achieving near-complete breakage within approximately 7 minutes (Supplementary Figure S1B). In contrast, the Balch system failed to efficiently disrupt J2s, likely due to their small size, and was excluded from subsequent optimization steps. These comparative tests highlight the critical importance of mechanical disruption in overcoming the nematode’s resistant cuticle of M. incognita. The Dounce homogenizer (pestle B) provided the most efficient and reproducible disruption. Although M. incognita shares structural features with C. elegans, such as a collagen-rich cuticle, its smaller size and tougher sheath require more finely controlled and gentle shear forces than more aggressive or larger-clearance systems [2,3]. These species-specific characteristics justify the adaptation of homogenization conditions.

**
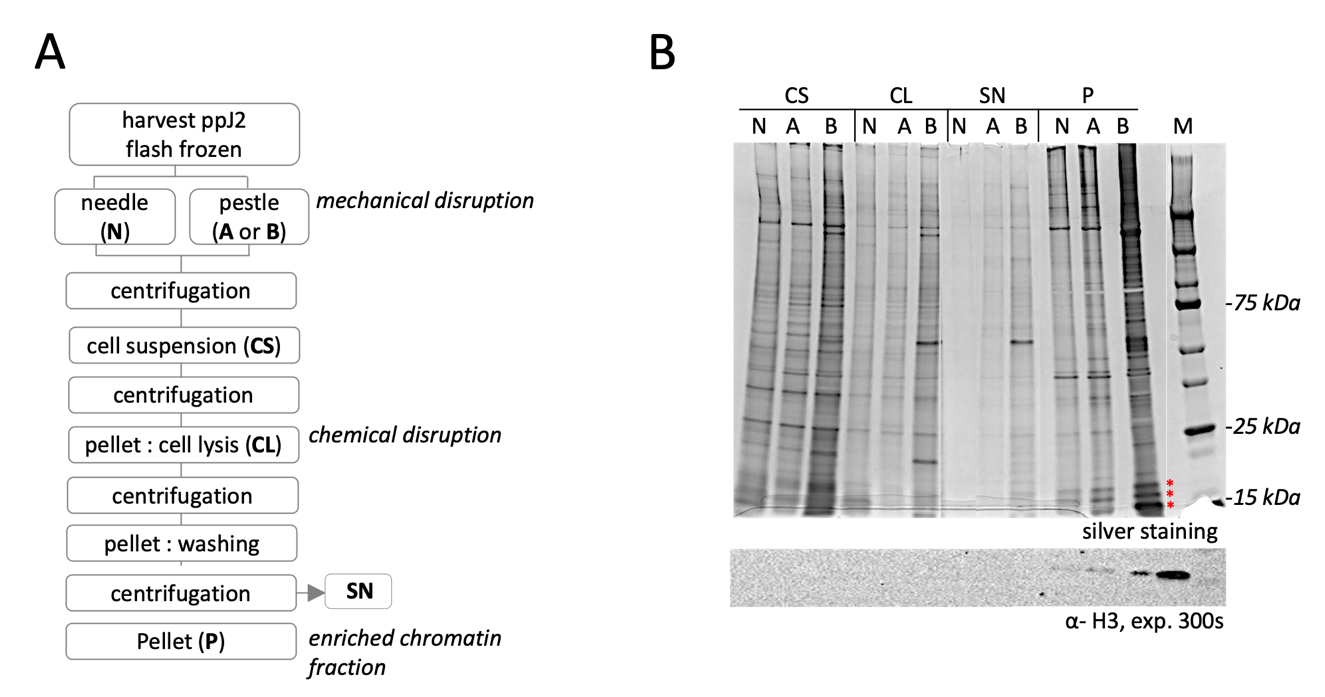
**

**Supplementary Figure S2.** **Comparison of homogenisation techniques for chromatin isolation from *M. incognita* J2 (A)** Schematic outlining the steps for chromatin isolation from frozen J2 using two manual homogenization devices: the syringe-needle system (N; 29-G diameter) and the 2 mL Dounce homogenizer (A “type loose“ and B “type tight”). Starting material consists of 30,000 J2 incubated in root extract for 2 hours, followed by flash-freezing in liquid nitrogen and storage at -80°C. Samples from each step were separated into supernatant (**CS, CL, SN**) and pellet (or nuclear crude extract, **P**) fractions. (**B**) SDS-PAGE (4-15%) separation of proteins from the different fractions. Each lane contains protein obtained by the preparation of 500 J2. Protein profiles at key stages of chromatin isolation revealed by silver staining (upper panel). Immunoblotting for histone H3 (bottom panel) highlights chromatin enrichment. Exposure time for chemiluminescence is indicated in seconds (s). Histone 3 presence (H3, ~16 kDA) is marked with red stars, confirmed by the anti-H3 antibody.

**Isolation of chromatin-rich fractions from nematode lysates -** After evaluating the impact of the different homogenization approaches on nematode body disruption, we next assessed their efficiency in recovering chromatin-associated proteins. The homogenization step was integrated into the chromatin extraction workflow following the Diagenode manufacturer’s guidelines. Ground J2 pellets were sequentially resuspended in the lysis buffers provided in the kit, and soluble fractions were collected after each centrifugation step (Supplementary Figure S2A). The final pellet, corresponding to the crude nuclear fraction, was analyzed by SDS-PAGE. Soluble fractions (SN) contained cytoplasmic and non-nuclear components, while the pellet (P) fraction was enriched in nuclear material, including chromatin-bound proteins. Silver-stained SDS-PAGE revealed substantial protein enrichment with distinct banding patterns in the crude nuclear fraction (P), with the most pronounced effect obtained using the Dounce homogenizer fitted with pestle B (Supplementary Figure S2B). The presence of histone proteins in this fraction was further supported by the appearance of characteristic bands on silver-stained gels and confirmed by immunoblotting against H3. These results demonstrate that mechanical disruption with the Dounce homogenizer (pestle B) is the most efficient to release chromatin-associated proteins while minimizing protein degradation. The accumulation of histone H3 in the nuclear-enriched fraction confirms the effectiveness of this step in preserving chromatin-bound material. Although the Dounce system consistently enriched the H3 chromatin marker more effectively than syringe-needle approach, the yield remained insufficient for downstream applications such as ChIP or dCas9-based CAPTURE assays.

*
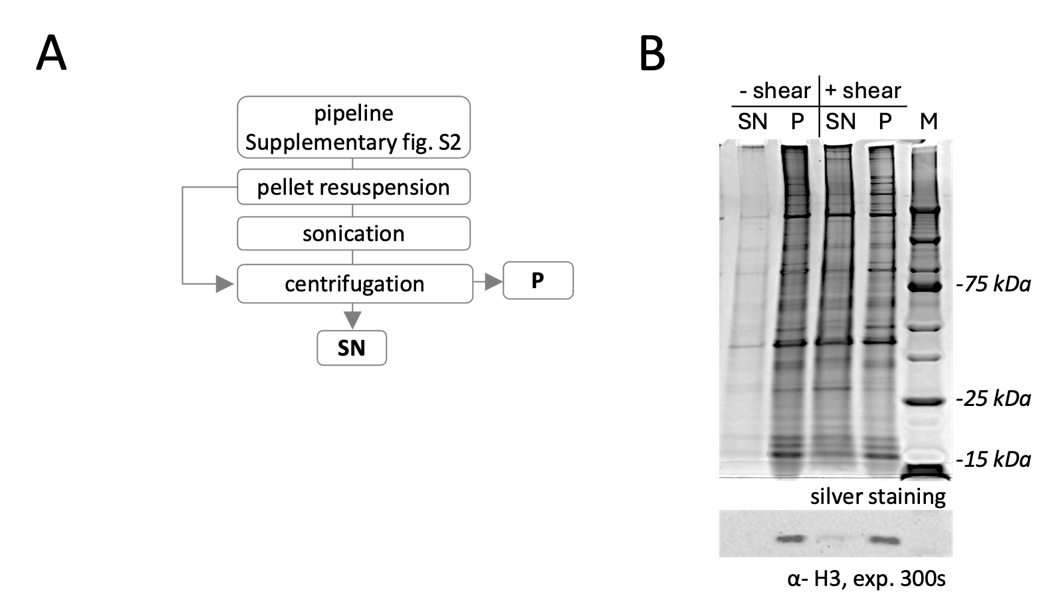
*

**Supplementary Figure S3. Impact of sonication on chromatin protein solubilization. (A)** Pipeline for chromatin isolation and shearing following dounce homogenizer with pestle B **(B)** Evaluation of chromatin shearing post-homogenization, where resuspended crude nuclear fractions were subjected to sonication or left untreated. Supernatant (SN) and pellet (P) fractions were collected and analyzed by 4-15% SDS-PAGE (upper panel). Silver staining shows an increase in solubilized proteins in the supernatant after sonication, confirming protein enrichment. Chromatin enrichment was validated by immunoblotting for Histone 3 (H3, ~16 kDa) (lower panel). The exposure time for chemiluminescence is indicated in seconds (s).

**Sonication improves chromatin protein solubilization, but DNA recovery requires further optimisations for ChIP applications** - To further optimize chromatin preparation, a sonication step was applied after dounce homogenization to shear chromatin into DNA fragments suitable for downstream ChIP assays (Supplementary Figure S3A). Following resuspension of the crude nuclear pellet (P), samples were either subjected to sonication of left untreated. Sonication facilitated the complete disaggregation of nematode material into smaller debris, as observed by microscopic examination. We then compared the protein profiles of the fractions, with and without sonication (Supplementary Figure S3B). Sonication increased solubilization of nuclear proteins, including H3, which appeared in the supernatant fraction (SN, + shear) using silver-stained SDS-PAGE (Supplementary Figure S3B; upper panel). However, histone H3 remained predominantly in the pellet (Supplementary Figure S3B; bottom panel). This suggests that the homogenization combined with biochemical treatments followed by the sonication step did not fully release nuclear proteins, leaving some chromatin trapped in the cellular debris. DNA recovery from 30,000 J2 yielded only ~100 ng . Using sonication alone, obtaining comparable DNA would require 50 times more material (~ 1 500,000 J2) – necessitating a much larger amount of infected plant material upstream to generate sufficient eggs and/or hatched J2. These demands increase when targeting multiple loci in dCas9-based CAPTURE assays. Our results underscore the need for further optimization of the sonication duration and intensity to maximize chromatin solubilization without compromising DNA integrity. This refinement ultimately guided the development of our “all-in-one” approach integrating mechanical disruption and chromatin shearing into a single optimized step.

**Material and methods**

**J2 sample preparation -** Aliquots containing approximately 30,000 M. incognita J2 or eggs, previously washed in Hank’s Balanced Salt solution buffer (HBSS, 5 mM KCl, 0.4 mM KH_2_PO_4_, 4 mM NaHCO_3_, 50 mM NaCl, 0.3 mM Na_2_HPO_4_, 5.5 mM Glucose; filtrated at 0.2 μm), centrifuged, and stored as pellets at −80 °C until use, were thawed on ice and briefly equilibrated to room temperature.

**Comparative manual homogenization and downstream chromatin quality assessment –** Approximatively 30,000 freshly collected second-stage juveniles (J2) or eggs were resuspended in 150 µL of cold supplemented with a freshly prepared 1X protease inhibitor cocktail (Roche, catalog # 11873580001). Samples were distributed into distinct handling devices for mechanical disruption: 1) Dounce Tissue Grinder (2-ml, Kimble kontes®) equipped with either the large-clearance pestle A (0.063 - 0.14mm, loose pestle) or the small-clearance pestle B (0.0127 - 0.0635mm, tight pestle), 2) Syringe fitted with a 29-gauge needle (29G, 0.33 mm x 13 mm) and 3) Balch homogenizer using different combinations of ball bearings i) 18 µm to 14 µm; ii) 14 to 6 µm and iii) 14 µm to 4 µm ball bearing, with 25 syringe passes per condition. Homogenization using the Balch system was performed in a hypotonic buffer (10 mM HEPES pH 7.6, 10 mM KCl, 1.5 mM EDTA, 250 mM sucrose). For reference, complete disruption of *C. elegans* L4 larvae – typically requires 25 passes with an 18 µL clearance ball bearing followed by 25 passes with a 12 µm clearance (ref 29, 30)

Manual disruption efficiency was monitored by optical microscopy on 5 μL of a 10-fold diluted J2 suspension. Resulting extracts were subsequently subjected to chemical lysis for chromatin isolation. Next steps were conducted at 4°C (centrifugation) or in a cold chamber room (incubation). Hereafter, all buffers from Diagenode Chromatin EasyShear kit - Low SDS (C01020013)) were freshly prepared on the day of use and supplemented with 1X protease inhibitors (Roche, catalog # 11873580001). J2 extracts were centrifuged at 2,700 x *g* for 5 min, and the pellet resuspended in 100 µL of cold lysis IL1b buffer for 10 min under gentle rotation (21 rpm). After centrifugation (2,400 rpm, 5 min.), the pellet was resuspended in 100 µL of cold lysis IL2 buffer for 10 min under the same conditions. Following another centrifugation step (2,400 rpm, 5 min), the pellet was resuspended in 100 µl of complete Shearing IS1 buffer and lastly centrifuged at 20,000 x *g* for 10 min. At this stage, the pellet fraction consisted predominantly of nuclear material, partially disrupted J2 debris and chromatin-rich aggregates, whereas supernatant contained soluble cytoplasmic proteins and other non-nuclear components. Samples were stored on ice for downstream quality control and quantification analyses, including agarose gel electrophoresis, bioanalyzer profiling, Qubit assays for DNA and SDS-PAGE and western blotting for protein content.

**Sonication treatment for chromatin fragmentation -** Following manual disruption with the Dounce homogenizer as described above, the resulting J2 pellets were resuspended in 100 µL of cold iS1 buffer supplemented with 1X protease inhibitors (Roche, catalog # 11873580001) and incubated 10 min. on a rotating wheel (21 rpm) in a cold chamber room. Samples were then transferred into 1.5 ml TPX microtubes (#M-50001, Diagenode) for sonication. Chromatin shearing was performed using a Diagenode Bioruptor PLUS instrument (#B01020004) under standard high-power settings (30 s ON/30 s OFF pulses for 10 cycles) at 4°C in a water bath without floating ice. Immediately after sonication, chromatin extracts were cleared in a hard spin at 16,000 x *g* for 10 min at 4°C. The resulting supernatants containing solubilized and sheared chromatin, were pooled, transferred to new 1.5 mL eppendorf tubes, and aliquoted for subsequent SDS-PAGE and Western blotting analyses. Chromatin extracts were snap-frozen in liquid nitrogen and stored at - 80°C for later use.

**Legends**

**Supplementary Figure S1.** Optimization of homogenization methods for the PPN *Meloidogyne incognita*. **(A)** Photos of the Balch homogenizer (left) and Dounce homogenizer (right). **(B)** Approximately 30,000 second-stage juveniles (J2) were resuspended in homemade Hank’s Balanced Salt solution (HBSS) buffer and gently disrupted on ice to generate a relative homogenate. Worm lysates were processed using various mechanical homogenization techniques: Dounce homogenizer with loose (A) or tight (B) pestle, Balch homogenizer using an 4- to 14 µm clearance ball bearing and with different combinations of syringe pass numbers, and Bioruptor device under different time and cycle conditions. All homogenization steps were performed on ice or at 4°C to maintain sample integrity. While these methods were effective in grinding nematode cells to subcellular components, none achieved complete homogenization. Further validation steps, including nuclear extract preparation, chromatin proteome analysis, and DNA isolation, were performed to ensure sample quality and compatibility for downstream analyses. Scale bar: 50µm

**Supplementary Figure S2.** **Comparison of homogenisation techniques for chromatin isolation from *M. incognita* J2 (A)** Schematic outlining the steps for chromatin isolation from frozen J2 using two manual homogenization devices: the syringe-needle system (N; 29-G diameter) and the 2 mL Dounce homogenizer (A “type loose“ and B “type tight”). Starting material consists of 30,000 J2 incubated in root extract for 2 hours, followed by flash-freezing in liquid nitrogen and storage at -80°C. Samples from each step were separated into supernatant (**CS, CL, SN**) and pellet (or nuclear crude extract, **P**) fractions. (**B**) SDS-PAGE (4-15%) separation of proteins from the different fractions. Each lane contains protein obtained by the preparation of 500 J2. Protein profiles at key stages of chromatin isolation revealed by silver staining (upper panel). Immunoblotting for Histone 3 (H3, bottom panel) highlights chromatin enrichment. Exposure time for chemiluminescence is indicated in seconds (s). H3 presence (~16 kDA) is marked with red stars, confirmed by the anti-H3 antibody.

**Supplementary Figure S3. Impact of sonication on chromatin protein solubilization. (A)** Pipeline for chromatin isolation and shearing following dounce homogenizer with pestle B **(B)** Evaluation of chromatin shearing post-homogenization, where resuspended crude nuclear fractions were subjected to sonication or left untreated. Supernatant (SN) and pellet (P) fractions were collected and analyzed by 4-15% SDS-PAGE (upper panel). Silver staining shows an increase in solubilized proteins in the supernatant after sonication, confirming protein enrichment. Chromatin enrichment was validated by immunoblotting for H3 (~16 kDa) (lower panel). The exposure time for chemiluminescence is indicated in seconds (s).

**References**

1. Hassanaly-Goulamhoussen R, De Carvalho Augusto R, Marteu-Garello N, Péré A, Favery B, Da Rocha M, Danchin EGJ, Abad P, Grunau C, Perfus-Barbeoch L. Chromatin Landscape Dynamics in the Early Development of the Plant Parasitic Nematode *Meloidogyne incognita*. Front Cell Dev Biol. 2021 Dec 6;9:765690. doi: 10.3389/fcell.2021.765690. PMID: 34938734; PMCID: PMC8685519.
2. Bhaskaran S, Butler JA, Becerra S, Fassio V, Girotti M, Rea SL. Breaking *Caenorhabditis elegans* the easy way using the Balch homogenizer: an old tool for a new application. Anal Biochem. 2011 Jun 15;413(2):123-32. doi: 10.1016/j.ab.2011.02.029. Epub 2011 Feb 24. PMID: 21354098; PMCID: PMC3521594.
3. Wibisono P, Liu Y, Sun J. A novel *in vitro Caenorhabditis elegans* transcription system. BMC Mol Cell Biol. 2020 Nov 30;21(1):87. doi: 10.1186/s12860-020-00332-8. PMID: 33256604; PMCID: PMC7706227.
4. Nouvel A, Laget J, Duranton F. et al. Optimization of RNA extraction methods from human metabolic tissue samples of the COMET biobank. Sci Rep 11, 20975. 2021. https://doi.org/10.1038/s41598-021-00355-x
